# Large language models in ophthalmology: promise, peril, and the urgent need for guardrails

**DOI:** 10.1038/s41433-026-04884-5

**Published:** 2026-09-17

**Authors:** Shameer Mohamed Naleer, Safras Mohamed Naleer

**Affiliations:** 1 Department of Surgery and Cancer, Imperial College London, London, UK; 2 Department of Biomedical Sciences, Brunel University of London, London, UK

**Keywords:** Outcomes research, Education

A registrar in a busy glaucoma clinic, running thirty minutes late, uses GPT-4 to draft a patient letter explaining a newly adjusted intra-ocular pressure target. The model produces fluent, empathetic prose—but cites a treatment threshold two points below current NICE guidance. The letter is sent unchecked. This is not a hypothetical: it is a failure mode already occurring in clinical settings, and one that current enthusiasm for large language models in ophthalmology has yet to confront seriously.

Large language models (LLMs) such as GPT-4, Gemini, and domain-specific variants are attracting significant attention across medicine for their potential to support clinical documentation, patient communication, and diagnostic triage. Ophthalmology is no exception: numerous publications have evaluated LLM performance on fellowship board examinations, patient-facing explanations of common diagnoses, and summarisation of clinical letters and medico-legal correspondence.

The performance of frontier LLMs on structured knowledge tasks is, in many cases, genuinely impressive. GPT-4 has been reported to achieve passing scores on ophthalmology fellowship examination questions, and to generate patient-facing explanations of diabetic retinopathy and glaucoma rated as comprehensible by non-specialist readers [1, 2]. This has generated enthusiasm about the potential of LLMs to alleviate documentation burden, patient education, and triage support in high-volume ophthalmic settings [3].

However, enthusiasm has outstripped governance. Three limitations demand attention before institutional adoption. First, LLMs lack real-time integration with clinical data—they cannot access a patient’s current visual acuity, intraocular pressure, or imaging results—and instead generate confident-sounding output based on statistical pattern-matching rather than clinical reasoning [4]. This is a significant limitation in a specialty where subtle imaging changes determine treatment decisions.

A diabetic patient’s risk stratification hinges on the exact extent and location of retinal neovascularisation [5]—information the LLM simply cannot access. Similarly, in glaucoma management, treatment escalation decisions depend on serial visual field progression and nerve fibre layer trends that exist only in the patient’s clinical record—not in the model’s training data [6]. In both cases, the confident output of an LLM is not anchored to the specific patient in front of the clinician.

Second, hallucination—the generation of plausible but factually incorrect information—remains an unresolved failure mode [7]. A 2023 analysis of LLM responses to ophthalmology patient queries found a significant proportion contained clinically significant inaccuracies, including incorrect dosing information and contraindications, posing real risk of harm to patients acting on these responses without clinical verification [8].

The same failure mode extends to ophthalmology-specific queries, an LLM asked to explain the management of acute angle-closure glaucoma may produce a response that is largely accurate but omits time-critical steps—such as the urgency of IOP lowering before definitive treatment—with potentially irreversible consequences for the patient. The risk is not that LLMs are frequently wrong—it is their errors are invisible, generated in the same fluent, authoritative register as their correct outputs [9].

Third, LLMs encode biases present in their training data. In ophthalmology, where diabetic retinopathy disproportionately affects South Asian populations, and glaucoma is more prevalent and severe in Afro-Caribbean patients, there is a genuine risk that LLM-generated guidance is systematically less accurate for under-represented groups [10]. The risk is not hypothetical: Gichova et al. demonstrated that retinal AI can infer patient race from fundus photographs without explicit training to do so, raising the possibility that LLMs trained on ophthalmic literature skewed toward high-income, majority-population datasets may produce substantially different outputs by demographic group—a pattern that would be invisible without disaggregated performance reporting [11].

The practical consequence in a UK NHS setting is not trivial, general practice surgeries in areas with large South Asian populations—precisely those carrying the highest burden of diabetes-related blindness—may have access to LLM tools whose performance may never have been evaluated against this patient group [10]. Until disaggregated performance reporting becomes standard practice, clinicians in these settings cannot know whether the tool they are using, performs equally for all their patients.

In the UK, NHS guidance on AI as a decision-support tool places clinical responsibility squarely with the supervising clinician—but provides no specific guidance on LLM use in documentation, patient communication, or triage [12]. The Royal College of Ophthalmologists has not yet issued LLM-specific guidance. This is particularly important because LLMs, unlike regulated medical devices, require no MHRA approval before clinical use: they are accessible to any clinician with internet access, without any systematic requirement for output validation, bias assessment, or incident reporting [13, 14].

This regulatory gap is not unique to the UK—however, it is particularly consequential in a publicly funded system where LLM adoption is likely to be driven by individual clinicians responding to administrative burden rather than by procurement processes with governance oversight built in.

We are not arguing against LLMs in ophthalmology—their potential to reduce administrative burden, enhance patient communication, and support non-specialist triage is real. We argue for governance to precede, not follow, deployment. Specifically, we call for mandatory auditing of LLM outputs against ground-truth clinical standards before institutional adoption; real-time disclosure to patients when AI tools have contributed to written communications; disaggregated performance reporting by demographic subgroup, to detect the differential accuracy that aggregate metrics conceal; and lastly, clear medico-legal frameworks defining liability when LLM-generated content results in harm.

For the RCOphth, issuing specialty-specific guidance on acceptable LLM use cases in ophthalmology—and explicitly excluded ones—would be a meaningful first step. In the interim, we propose a minimum standard for institutional LLM use in ophthalmic settings: all patient-facing communications generated with LLM assistance should be reviewed and countersigned by a qualified clinician; all LLM outputs should be flagged as AI-assisted in the clinical record; and any suspected LLM error contributing to a patient safety incident should be reportable through existing incident reporting frameworks [15]. These requirements impose no new infrastructure—they require only that LLMs be treated with the same oversight applied to any clinical decision-support tool.

LLM development has outpaces ophthalmic governance, that gap is not optional; it is a patient safety imperative—and the registrar who sent that letter is still waiting to find out.
